# Newly discovered genomic mutation patterns in radiation-induced small intestinal tumors of *Apc*^*Min/+*^ mice

**DOI:** 10.1371/journal.pone.0292643

**Published:** 2023-10-12

**Authors:** Daisuke Iizuka, Megumi Sasatani, Atsuko Ishikawa, Kazuhiro Daino, Tokuhisa Hirouchi, Kenji Kamiya

**Affiliations:** 1 Department of Radiation Effects Research, Institute for Radiological Science, National Institutes for Quantum Science and Technology, Chiba, Japan; 2 Department of Experimental Oncology, Research Institute for Radiation Biology and Medicine, Hiroshima University, Hiroshima, Japan; 3 Department of Radiobiology, Institute for Environmental Sciences, Rokkasho, Japan; King Faisal Specialist Hospital and Research Center, SAUDI ARABIA

## Abstract

Among the small intestinal tumors that occur in irradiated mice of the established mouse model B6/B6-Chr18^MSM^-F1 *Apc*^*Min/+*^, loss of heterozygosity analysis can be utilized to estimate whether a deletion in the wild-type allele containing the *Adenomatous polyposis coli* (*Apc*) region (hereafter referred to as Deletion), a duplication in the mutant allele with a nonsense mutation at codon 850 of *Apc* (Duplication), or no aberration (Unidentified) has occurred. Previous research has revealed that the number of Unidentified tumors tends to increase with the radiation dose. In the present study, we investigated the molecular mechanisms underlying the development of an Unidentified tumor type in response to radiation exposure. The mRNA expression levels of *Apc* were significantly lower in Unidentified tumors than in normal tissues. We focused on epigenetic suppression as the mechanism underlying this decreased expression; however, hypermethylation of the *Apc* promoter region was not observed. To investigate whether deletions occur that cannot be captured by loss of heterozygosity analysis, we analyzed chromosome 18 using a customized array comparative genomic hybridization approach designed to detect copy-number changes in chromosome 18. However, the copy number of the *Apc* region was not altered in Unidentified tumors. Finally, gene mutation analysis of the *Apc* region using next-generation sequencing suggested the existence of a small deletion (approximately 3.5 kbp) in an Unidentified tumor from a mouse in the irradiated group. Furthermore, nonsense and frameshift mutations in *Apc* were found in approximately 30% of the Unidentified tumors analyzed. These results suggest that radiation-induced Unidentified tumors arise mainly due to decreased *Apc* expression of an unknown regulatory mechanism that does not depend on promoter hypermethylation, and that some tumors may result from nonsense mutations which are as-yet undefined point mutations.

## Introduction

Ionizing radiation is classified as a carcinogen, as evidenced by epidemiological studies of atomic bomb survivors [1]. Even though, it is considered a weak carcinogen compared with chemical carcinogens [2]. As radiation carcinogenesis is thought to originate from genomic abnormalities caused by radiation exposure, much research effort has been directed towards understanding the genomic mutations that are characteristic of radiation-induced tumors. Genomic abnormalities related to radiation carcinogenesis, such as *RET/PTC* translocation, have been observed in genomic analyses of thyroid tumors associated with radiation exposure in humans [3]. However, no such genomic abnormalities have been found in other tumor types associated with radiation exposure.

Identifying the signatures of ionizing radiation in human tumors is challenging because cancers can result from a combination of various causes, including ionizing radiation, smoking, diet, and ultraviolet radiation. Therefore, animal models are important for investigating the underlying mechanisms of radiation carcinogenesis. Upon loss of heterozygosity (LOH), mutations are more likely to occur in the other normal allele, which is one of the mechanisms underlying the inactivation of certain tumor suppressor genes. Therefore, the use of experimental animals carrying mutations in one allele of a tumor suppressor gene has led to dramatic progress in carcinogenesis research. Recently, it was reported that, in *Ptch1* heterozygous (*Ptch1*^*+/–*^) mice of the medulloblastoma tumor model and Eker (Long Evans *Tsc2*^*+/–*^) rats of the renal cancer model, LOH analysis can be used to estimate whether deletion of a wild-type allele containing a cancer-causing gene region (Deletion), duplication of a mutant allele with a mutation in a cancer-causing gene occurred (Duplication), or no abnormality (Unidentified) occurred, and that the Deletion type is increased in a dose-dependent manner [4–6]. Radiation exposure causes DNA double-strand breaks, and cells in which tumor suppressor gene(s) are deleted (as a consequence of the DNA repair response) may be the primary driver of tumorigenesis [2].

*Apc*^*Min/+*^ (multiple intestinal neoplasia) mice, originally generated via random mutagenesis using *N*-ethyl-*N*-nitrosourea (ENU), are an experimental model of human familial adenomatous polyposis [7]. *Apc*^*Min/+*^ mice can spontaneously develop intestinal adenomas, and therefore constitute a useful animal model for studying the effects of radiation and chemical carcinogens on the development of intestinal tumors. *Apc*^*Min/+*^ mice carry a heterozygous germline nonsense mutation in codon 850 of *Apc* on chromosome 18 (Chr18), which results in a truncated, non-functional Apc protein. Inactivation of the remaining normal *Apc* allele leads to spontaneous development of multiple intestinal tumors. By using the B6-Chr18^MSM^ consomic mouse strain in which Chr18 (and therefore *Apc*) of mouse strain B6 is replaced with a corresponding chromosome of strain MSM/Ms (MSM). We previously showed that deletion of the wild-type allele containing the *Apc* region (Deletion) or duplication of the *Min* allele (Duplication) can be detected by LOH analysis of radiation-induced small intestinal tumors in B6/B6-Chr18^MSM^-F1 *Apc*^*Min/+*^ mice. We found an age-at-exposure-dependent association between the induction of small intestinal tumors and an increase of the Deletion type [8]. In addition, we identified tumors without LOH (Unidentified) that tended to occur more frequently in irradiated juvenile mice [8]. We also found that the incidence of these Unidentified tumors tended to increase with the radiation dose (Sasatani et al, in preparation). Two possible mechanisms for this are that radiation exposure increases methylation of the *Apc* promoter region with an accompanying decrease in *Apc* expression, or accelerated mutations in *Apc* with consequent dysfunction of the Apc protein. It is also possible that the number of small deletions that cannot be determined by LOH is greater in Unidentified tumors; however, this is strictly classified as the Deletion type. Therefore, we investigated the mechanism underlying the increase in the number of radiation-induced Unidentified tumors by examining their characteristics.

## Materials and methods

### Animal experiments and gamma irradiation

Mouse experiments and gamma irradiation were performed as described previously [8]. Briefly, male, or female B6/B6-Chr18^MSM^-F1 *Apc*^*Min/+*^ mice were obtained by crossing female B6 *Apc*^*Min/+*^ mice with male B6-Chr18^MSM^ mice. Gamma irradiation was performed using a Gammacell 40 Exactor (Best Theratronics, Ottawa, Canada). The mice were placed in a plastic irradiation cage without anesthesia, and irradiation was completed as quickly as possible. The mice were gamma-irradiated at 2 weeks of age, after which their condition was observed at least twice a week, and they were euthanized with isoflurane anesthesia at 24 weeks of age. Although cases of immobility, anemia, and intestinal bleeding as indicators of humane endpoints are rare, if such symptoms were observed, the animals were euthanized as described above. At the time of dissection, almost all tumors were collected from the entire small intestine under a stereomicroscope, frozen in liquid nitrogen, and stored at –80 °C. All experiments with mice were conducted in accordance with the principles and procedures outlined in the protocols of the authors’ institution after authorization by the Institutional Animal Care and Use Committee of the Hiroshima University (authorization number: A22-45).

### DNA and RNA extraction and qRT-PCR analysis

DNA and RNA were extracted from cryopreserved tumor tissues using the AllPrep DNA/RNA Micro kit (Qiagen, Hilden, Germany) according to the manufacturer’s instructions. Quantitative reverse transcription (qRT)-PCR for *Apc* was carried out using SYBR Green (Thunderbird SYBR qPCR Mix; TOYOBO, Osaka, Japan) as described previously [9] using a LightCycler96 Real-time PCR System (Roche Diagnostics, Mannheim, Germany) with the specific primers listed in S1 Table. Expression of mouse *Gapdh* (encoding glyceraldehyde 3-phosphate dehydrogenase) was used as an internal standard for calculating relative gene expression levels with the 2^–ΔΔCT^ method [10].

### Sodium bisulfite DNA sequencing in the *Apc* promoter region

The methylation state of the *Apc* promoter region was determined by sodium bisulfite treatment of either 1000 ng of genomic DNA with the CpGenome Fast DNA Modification kit or 80–300 ng of genomic DNA with the CpGenome Turbo DNA Modification kit (Merck Millipore, Burlington, MA), after which the nucleotide sequence was determined according to the manufacturer’s instructions. Because it was difficult to amplify the entire CpG island within the *Apc* promoter with one primer set, two primer sets were used (hereafter referred to as CpG islands 1 and 2) (S1 Fig). Using bisulfite-treated DNA as a template, Ex Taq DNA polymerase (Takara Bio Inc., Shiga, Japan) was used to amplify CpG island 1, and EpiTaq HS (Takara Bio Inc.) was used to amplify CpG island 2. The reaction conditions are shown in S1 Table. The PCR product was cloned into pCR2.1-TOPO vectors using the TA cloning kit (TOPO TA Cloning H kit, Invitrogen, Carlsbad, CA). An ABI PRISM Dye Deoxy Terminator Cycle Sequencing Kit and an ABI 3500xl DNA Sequencer (Applied Biosystems, Foster City, CA) were used to determine the CpG methylation status of the *Apc* promoter region, and at least four clones per tumor were sequenced.

### DNA copy-number analysis

The comparative genomic hybridization (CGH) array used in this study was the Mouse Genome CGH Microarray (Agilent Technologies, Santa Clara, CA) 8X60 k format custom microarray (GPL32325) (Protocol version 7.3). A custom array was created using Agilent’s custom array creation tool (eArray) according to the mm9 mouse genomic database. Briefly, the genomic DNA from normal small-intestinal tissue of six males and one female, pooled in equal amounts, was used for hybridization. Tumor DNA (200 ng) and the reference DNA were labeled with Cy5- and Cy3-dUTP, respectively (Agilent Technologies). After hybridization of the labeled and microarray DNA, fluorescence intensity data were obtained using a DNA microarray scanner (Agilent Technologies) according to the manufacturer’s recommended settings and quantified using Agilent Feature Extraction software (version 10.5.1.1). Statistical analysis was performed using Genomic Workbench software (version 7.0.4; Agilent Technologies). Statistically significant copy number changes were determined (range, 6.0) using the Aberration Detection Method-2 (ADM-2) algorithm. Microarray data were deposited in the Gene Expression Omnibus database (www.ncbi.nlm.nih.gov/geo; accession No. GSE206669).

### DNA hybridization and sequencing

The amount of DNA used was determined by quantifying double-stranded DNA using a Qubit 2.0 fluorometer (Thermo Fisher Scientific, Waltham MA). DNA (66 ng) was enzymatically fragmented using the SureSelect Enzymatic Fragmentation kit (Agilent Technologies). A DNA library was created using a SureSelect XT HS Library Preparation Kit. The SureSelect custom probe was designed for the mouse *Apc* region (Chr18:34220984–34322552, region size 101.569 kbp; UCSC mm10, GRCm38, December 2011) (https://earray.chem.agilent.com/suredesign/, Agilent Technologies). The total number of probes was 2994, the total probe size was 82.210 kbp, and exon coverage was 100% (total coverage was ~93.9%). Using a custom probe, the library was hybridized to the SureSelect XT HS Target Enrichment kit. Hybridized DNA was captured using streptavidin-coated beads and PCR-amplified to prepare SureSelect-enriched, indexed, molecular-barcoded next-generation sequencing samples. These libraries were sequenced using the 75-bp paired-end protocol on a MiSeq sequencer (Illumina, San Diego, CA).

### Next-generation sequencing data analysis

Somatic mutations were detected using a pipeline developed by Amelieff Co. Ltd. (Tokyo, Japan) as previously described, with minor modifications [11]. Briefly, the reads were trimmed by removing low-quality bases, which were removed if they were shorter than 32 bases or if more than 80% of any individual read had a quality rating of 20 or less using the QCleaner tool, and then aligned to the mouse reference genome (GRCm38) using the Burrows-Wheeler Alignment tool (version 0.7.16a). Duplicate reads were removed using SAMtools (version 1.6), and base quality recalibration and realignment around insertions/deletions were performed using the Genome Analysis Tool Kit (version 1.6–13). Somatic single-nucleotide variants and insertions/deletions in tumors were called using VarScan 2 software (version 2.4.3) [12]. A false positive filter was applied to remove sequencing- or alignment-related artifacts. Variants were annotated, and their effect on coding sequences was predicted using SnpEff software (version 4.3) [13]. We also required that there be no representations of normal reads. Control-FREEC software was used to identify copy number changes in tumors compared to normal tissues [14]. Segments with statistically significant copy number changes compared with normal tissues were extracted. Exclusion criteria were as follows: 1) not a somatic mutation, 2) present in normal tissue, 3) not statistically significant (p > 0.05, Wilcoxon and Kolmogorov-Smirnov tests), and 4) regions containing repeat sequences determined by IGV software (Broad Institute, Cambridge, MA).

### Statistical analyses

Results are presented as the mean ± SD. Differences between groups were evaluated by one-way ANOVA followed by Tukey’s test using GraphPad Prism software (version 8.4.3), and values of p < 0.05 were considered statistically significant.

## Results

### *Apc* expression is decreased in Unidentified type tumors

**Fig 1:**
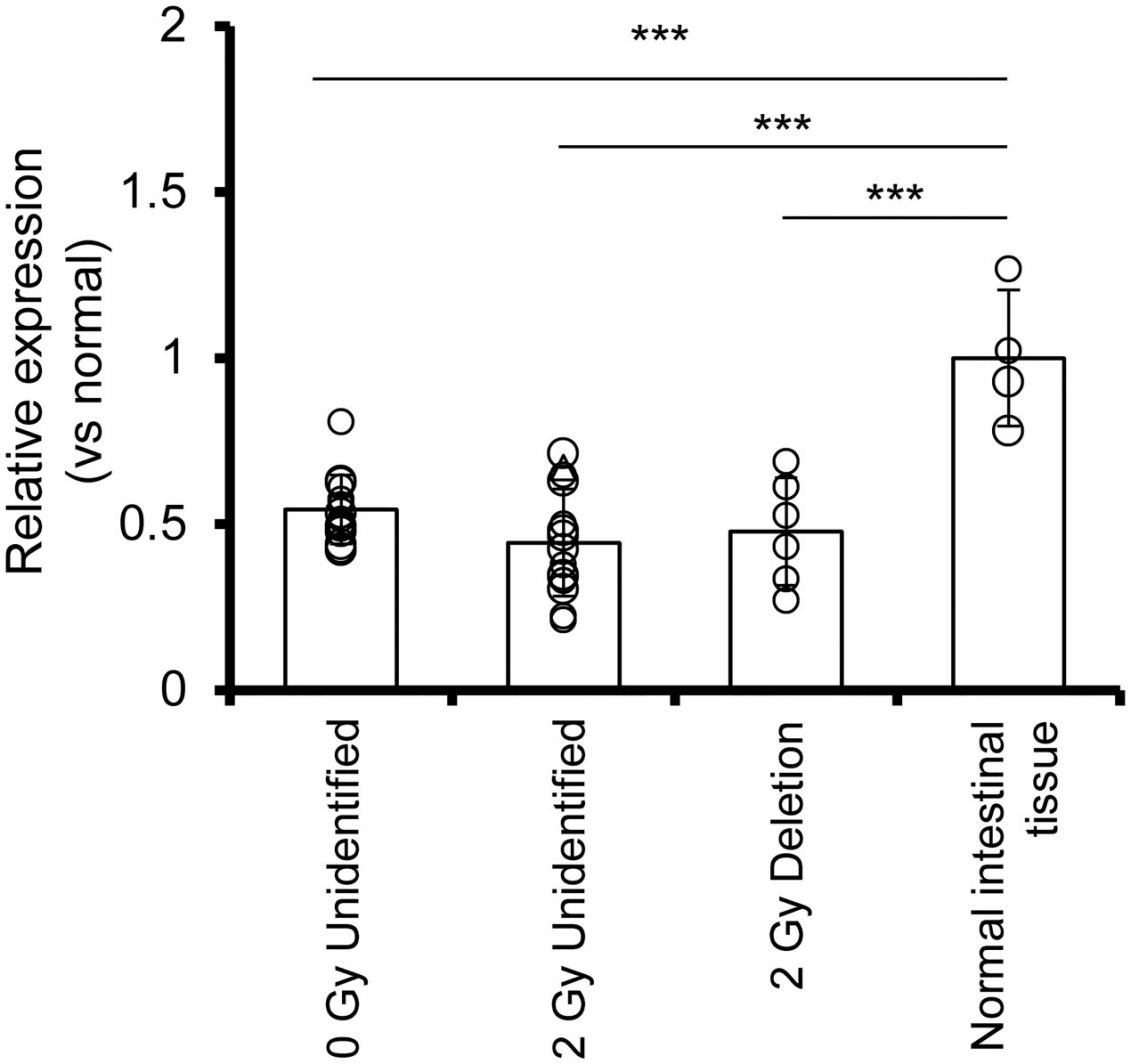
Analysis of *Apc* mRNA expression in small intestinal tumors of B6/B6-Chr18^MSM^-F1 *Apc*^*Min/+*^ mice. Quantitative PCR was performed using *Gapdh* as an internal control. Each point represents *Apc* mRNA level in an individual tumor (0 Gy Unidentified, n = 14; 2 Gy Unidentified, n = 15; 2 Gy Deletion, n = 6; normal intestinal tissue, n = 4). Primers were set to span exons 2 and 3 according to the NCBI database (NM_001402727.1). The means and standard deviations are represented by bars and error bars, respectively. *Apc* expression in normal intestinal tissue was set to 1. The triangle among the 2 Gy-Unidentified data points represents *Apc* expression in Tumor ID 2-2-23, in which a deletion comprised approximately 3.5 kbp at the end of the last exon of *Apc*. *** p < 0.001 versus normal intestinal tissue group.

We previously reported that in B6/B6-Chr18^MSM^-F1 *Apc*^*Min/+*^ mice, small intestinal tumors increased in an age-at-exposure-dependent manner [8], and that the proportions of both Unidentified and Deletion-type tumors increased with radiation dose (Sasatani *et al*, in preparation). The mean diameter (mm) of the tumors analyzed in this study and their 95% confidence intervals were 1.68 (1.47, 1.89) and 1.63 (1.48, 1.79) for the 0 Gy and 2 Gy exposure groups, respectively. The maximum diameter of the tumors was 3.47 mm and 3.58 mm in the 0 Gy and 2 Gy exposure groups, respectively. S2 Fig shows the LOH patterns of the tumors analyzed in this study. As Unidentified tumors may have reduced *Apc* mRNA expression due to hypermethylation of the *Apc* promoter region, we first quantified *Apc* mRNA levels using RT-qPCR. *Apc* expression was significantly reduced by approximately one-half in both Unidentified and Deletion-type tumors compared to normal tissues (Fig 1). This reduction was similar when different exons of *Apc* were examined (S3 Fig).

### Methylation of the *Apc* promoter region does not occur in Unidentified type tumors

**Fig 2:**
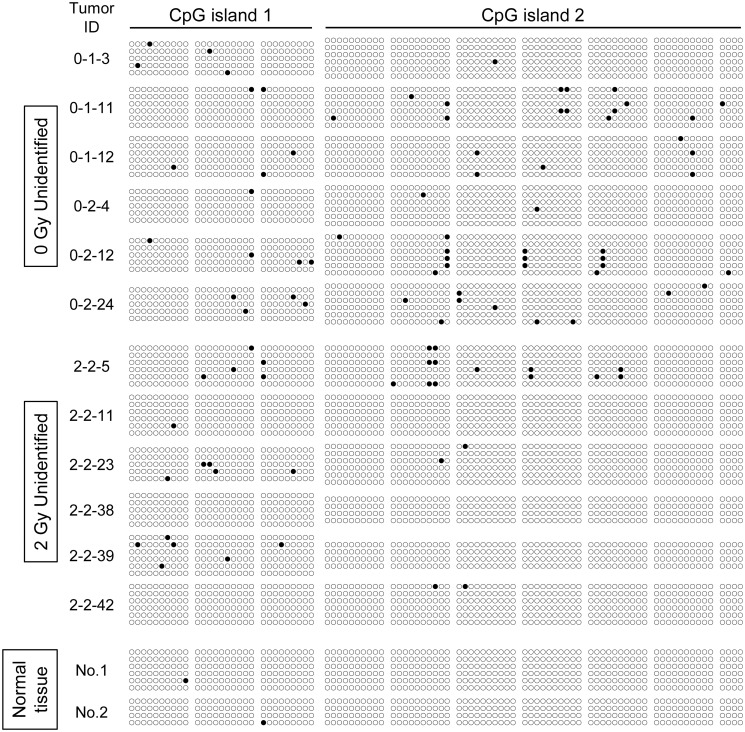
Bisulfite sequencing of the *Apc* promoter region in small intestinal tumors of B6/B6-Chr18^MSM^-F1 *Apc*^*Min/+*^ mice. Methylation status of the 93 CpG dinucleotides in the promoter region of *Apc*. Each circle represents a CpG site in the genomic DNA sequence and each row of circles represents the analysis of a single cloned allele. Closed circles: methylated CpG dinucleotides; open circles: unmethylated CpG dinucleotides. Bisulfite efficiency averaged 98.5 ± 1.33% for CpG island 1 and 98.8 ± 1.23% for CpG island 2 in all samples examined.

We hypothesized that the decreased level of *Apc* mRNA in Unidentified tumors is attributed to an epigenetic regulatory mechanism; therefore, we analyzed the methylation status of the *Apc* promoter region using a sequence of bisulfite-treated DNA. We first assessed the *Apc* region (18qB1; 34220984–34322190, NM_007462, GRCm38 / mm10 assembly) using the UCSC Genome Browser, which revealed that CpG islands with abundant CG sequences were present around the transcription start site (S1 Fig). Bisulfite sequencing revealed that most of the 93 cytosines that could potentially be methylated were not (Fig 2). These results indicated that the *Apc* promoter region was not methylated in most cells in the Unidentified tumors.

### *Apc* deletions are predominant in Deletion type but not Unidentified type tumors

**Fig 3:**
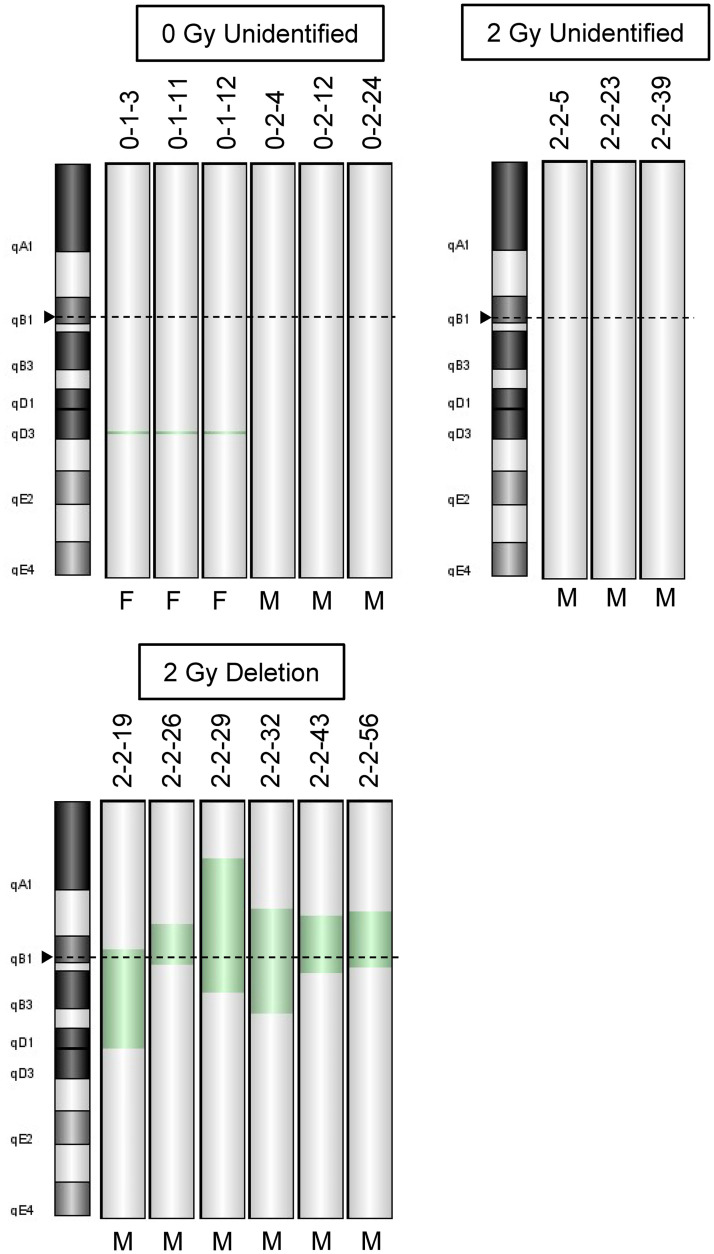
Array CGH analysis of small intestinal tumors of B6/B6-Chr18^MSM^-F1 *Apc*^*Min/+*^ mice. The results for Chr18 are shown for each tumor type. The Tumor ID is shown above each depicted Chr18, and the sex of each mouse in which the tumor arose is indicated below (M, male; F, female). Green indicates a deletion locus. Arrowheads and corresponding dashed lines indicate the *Apc* locus.

As *Apc* expression is regulated by mechanisms other than promoter methylation, we considered accelerated degradation of *Apc* mRNA due to genetic mutations a possible mechanism. Because our LOH analysis revealed the LOH of microsatellite DNA, even though LOH between marker sites was not demonstrated, we investigated DNA copy number aberrations using array CGH. For Chr18, where *Apc* is located, a custom microarray CGH was used, in which the corresponding DNA probes were designed at intervals of ~1.5 kbp, that is, there were 65 probes for the *Apc* region (total length ~100 kbp). As expected, deletions of *Apc* were observed in Deletion type tumors (mean deletion size; 17,769.9 ± 7918.0 kbp, Fig 3). These deletions were consistent with the observations from the LOH analysis (S2 Fig). For Unidentified tumors, nine tumors, that is, six from the non-irradiated group and three from the 2 Gy exposure group, were analyzed; however, no genomic copy number aberration exceeding the threshold was apparent in the genomic region where *Apc* was located (Fig 3, S2 Table). Of the Unidentified tumors from the non-irradiated group, region 18qB1 was deleted in Tumor IDs 0-1-3, 0-1-11, and 0-1-12 (mean deletion size; 47 ± 0.87 kbp); this region contains part of the gene *Slc27a6*. Abnormalities in the X-chromosome copy number were observed depending on the sex of the individual in which the tumor arose (S2 Table).

### Nonsense and frameshift *APC* mutations have been detected in some Unidentified tumors

Next, detailed *Apc* copy number aberration analysis was performed using next-generation sequencing. In addition to the tumors analyzed by the CGH array, 13 tumors were subjected to next-generation sequencing. A specific capture probe was designed and the sequence of the *Apc* region was determined. Among the extracted results, the data mapped to regions other than Chr18 were omitted. Analysis of *Apc* copy number aberrations revealed a deletion of approximately 3.5 kbp (from Chr18:34,313,764 to Chr18:34,317,250) in the coding region at the end of the last exon in a single tumor (Tumor ID 2-2-23). Using GA nucleotide sequences, the mode of recombination in this region was inferred to be microhomology-mediated end-joining.

Finally, somatic mutations were examined. Table 1 shows the frequency of the mutant alleles of 5% or more. Nonsense or frameshift mutations due to base substitutions or single-nucleotide deletions were found in four Unidentified tumors (Tumor IDs: 0-1-12, 0-2-24, 2-2-39, and 2-2-42) in both the non-irradiated and 2 Gy exposure groups (Table 1). However, in other tumors, no mutations were found in the exons or intron-splice acceptor/donor sites within the *Apc* region (S3 Table). These results suggested that the reduced *Apc* expression observed in radiation-induced Unidentified tumors was caused, at least in part, by a small deletion in the *Apc* locus or nonsense mutations.

**Table 1:** Mutations identified in the Unidentified type of intestinal tumors.

	Tumor ID	Position in Chr.18	Ref. Seq	Alt. Seq	Variant allele frequency (%)	HGVSc^†^	Annotation	HGVSp^‡^	Exon affected
0 Gy Unidentified	0-1-12	34298758	G	A	6.39	c.1263G>A	stop_gained	p.Trp421*	exon 10
	0-2-24	34312033	C	T	30.39	c.1981C>T	stop_gained	p.Gln661*	exon 16
2 Gy Unidentified	2-2-39	34312841	C	G	21.59	c.2789C>G	stop_gained	p.Ser930*	exon 16
	2-2-42	34279314	GT	G	21.3	c.686delT	frameshift_variant	p.Val229fs	exon 7

^†^HGVS (Human Genome Variation Society) coding sequence name

^‡^HGVS protein sequence name

## Discussion

In this study, we investigated the molecular mechanisms underlying radiation-induced Unidentified tumors from both genetic and epigenetic perspectives. The results showed that *Apc* mRNA expression was significantly decreased in Unidentified tumors compared to that in normal tissues. We focused on epigenetic suppression as the mechanism underlying this decreased expression; however, hypermethylation of the *Apc* promoter region was not observed. Array comparative genomic hybridization analysis and next-generation sequencing revealed no copy number aberrations in the *Apc* region except in one tumor; however, *Apc* nonsense and frameshift mutations were observed. These results suggest that radiation-induced Unidentified tumors arise mainly due to decreased *Apc* expression of an unknown regulatory mechanism that does not depend on its promoter hypermethylation, and that some tumors may result from nonsense mutations which are as-yet undefined point mutations.

For the first time, we performed DNA sequencing of *Apc* in Unidentified tumors of B6/B6-Chr18^MSM^-F1 *Apc*^*Min/+*^ mice. As a result, nonsense or frameshift mutations were observed in four tumors. Similar to *Ptch1*^*+/–*^mice (medulloblastoma model) and Eker rats (renal cancer model), some Unidentified tumors in *Apc*^*Min/+*^ mice could not be identified by sequence analysis [4–6]. In addition, medulloblastoma in *Ptch1*^*+/–*^mice and renal cancer in Eker rats are caused by nonsense mutations associated with deletions spanning from several to hundreds of bases. In contrast, the nonsense mutations that occurred in *Apc* were mainly point mutations rather than deletions. This suggests that mutations in the causative gene occur in a tumor- or gene-specific manner. In addition, radiation-induced Unidentified tumors may have contained a small deletion that could not be detected by LOH analysis. There were no other significant differences from the non-exposed group in this study. Recently, it was proposed that radiation carcinogenesis may be an indirect consequence of inflammation (i.e., radiation exposure) in the tissue microenvironment [15]. Radiation carcinogenesis may have been caused by such a mechanism, as some radiation-induced gene mutations were not detected in some tumors in the present study.

More than 60% of human *APC* mutations are located in the mutation cluster region (human codons 1286–1513) in exon 15 [16], and in most cases, a mutated *APC* expresses a truncated protein [17]. In addition, a truncation mutation in ENU-induced tumors in *Apc*^*Min/+*^ mice was found in codon 971–1197 (ENU mutation cluster region) [18]. The sites of the nonsense mutations and the missense frameshift variants identified in this study did not match these ENU-mutation cluster regions. These results indicate that Unidentified tumors, regardless of radiation exposure, have a mutation profile different from that of tumors induced by chemical carcinogens.

Approximately 70% of the mutations observed in this sequencing analysis had low variant allele frequencies, or were intron variants with little biological significance (Table 1 and S3 Table). One of the reasons for the low overall frequency of mutant alleles is that the tumors were collected under a stereomicroscope. Mutations can be detected more accurately by collecting only the tumor site via laser microdissection and extracting its DNA. Several of these mutations have been identified at the splice acceptor/donor sites of *APC* [19], however they have not been detected in the tumor samples in this study. Some of the mutations detected in this study have relatively high variant allele frequencies; therefore, intron mutations may play a role in the carcinogenesis of Unidentified tumors.

In this study, no change in DNA copy number was detected in Unidentified tumors, even with a custom microarray CGH, in which a DNA probe was attached at a high density to Chr18. In contrast, partial deletion of the *Slc27a6* region was observed in some Unidentified tumors. *Slc27a6* encodes a fatty acid transporter. Long-chain fatty acids are involved in various physiological processes, such as inflammation and phospholipid and triglyceride synthesis [20, 21]. This transporter is associated with the regulation of cancer cell behavior. It has been reported that *Slc27a6* expression is downregulated in breast cancers, but this is the first finding in intestinal tumors [22]. In our study, the deletion of the *Slc27a6* region was observed in only three tumors arising in females. As sex differences in genomic copy number aberrations have been reported in several human tumors [23], this mouse model also suggests sex differences in small intestinal tumorigenesis. These abnormalities may be partly responsible for the mechanism of small intestinal tumor development, which is independent of the acquisition of stop codons due to partial deletions in *Apc* or nonsense mutations. Further investigation is required to support this hypothesis.

Most of the tumor samples evaluated were free of *Apc* mutations. This result was unexpected because carcinogenesis in this model is associated with either the loss or inactivation of the wild-type allele, according to Knudson’s two-hit theory [24]. However, *Apc* expression is downregulated in most tumors. The nonsense mutations identified in this study were essentially ineffective in nonsense-mediated mRNA decay. However, it has been suggested that sequences downstream of the nonsense mutation in the final exon may behave as a 3’ untranslated region, and that microRNAs binding to such sequences may cause downregulation of *Apc* [25]. In addition, epigenetic changes have been suggested to be involved in the inactivation of the *Apc* wild-type allele. Haigis *et al* reported in an LOH analysis that the wild-type *Apc* allele was not deleted in small intestinal tumors in *Apc*^*1638N/+*^ mice. The authors concluded that epigenetic changes are involved in the development of small intestinal tumors [26]. However, in the present study, only minimal methylation of the *Apc* promoter region was observed. The presence of two promoters, 1A and 1 B, has been reported in human *APC* [27]. Abnormal methylation in the 1A region has been reported in human breast and lung cancers [28]. In mice, there are sites corresponding to the human 1A region, but the 21 CpG sites are also mostly unmethylated (S4 Fig). Changes in small RNA and histone modifications are considered epigenetic regulatory mechanisms, in addition to DNA methylation in the promoter region. Regarding the regulatory mechanism of miRNAs, the miR-135 family (miR-135a and b) was found to be upregulated in human colorectal adenomas and colorectal cancers and was associated with low expression of the *Apc* [29]. This regulatory mechanism has been shown to be unrelated to mutations in *Apc*. Abnormalities in histone modifications or long noncoding RNA expression are known to cause intestinal tumors; however, no evidence has been reported that directly regulates *Apc* expression. Based on these results, there may be unknown mechanisms for suppressing *Apc* expression via mutations in factors that regulate *Apc* expression rather than direct mutations in *Apc*. Further investigation is required to clarify the mechanism underlying such downregulation.

This study examined the molecular mechanisms underlying radiation-induced Unidentified tumors from both genetic and epigenetic perspectives. However, this study had several limitations. As mentioned above, the tumors obtained in this study were small; therefore, it was challenging to perform both genetic and histopathological analyses of a single tumor. Therefore, it was not possible to evaluate the tumor grade. Morioka et al. reported the tumor diameter and grading in C3B6F1 *Apc*^*Min/+*^ mice [30]. The resulting small-intestinal tumors were classified as adenomas and adenocarcinomas, with adenocarcinomas being significantly larger in diameter than adenomas, which did not change with irradiation. The results showed that the average diameter of the adenomas was < 2 mm, whereas that of the adenocarcinomas was greater than 3 mm. Thus, it can be inferred that most of the tumors obtained in this study were adenomas and that irradiation did not change the size or tumor grading. Second, it was not possible to analyze APC protein expression levels. The small size of the tumors obtained in this experiment made it difficult to simultaneously perform immunostaining and western blot analysis, in addition to genetic analysis of a single tumor. It is also worth mentioning that the detection of mouse APC has rarely been reported. Previous studies on genetically engineered Apc mice have shown that mutations of amino acid 1309 at the N-terminal of the APC protein play an important role in tumorigenesis [31], suggesting the importance of the novel mutation found in this study in tumorigenesis. Further analysis is needed to unveil how the nonsense and frameshift mutations detected in this study affect the function of the APC protein, and how frequently these mutations are induced in tumorigenesis. These are essential issues to be analyzed in future studies on tumors arising in B6/B6-Chr18^MSM^-F1 *Apc*^*Min/+*^ mice.

In summary, this study clarified a part of the mechanism of radiation-induced Unidentified tumors in B6/B6-Chr18^MSM^-F1 *Apc*^*Min/+*^ mice. As the investigation of Unidentified tumors progresses in the future, it is expected that a full picture will arise of the mechanism of small intestinal tumor induction, including induction by radiation exposure.

## Supporting information

S1 Table Primers and PCR conditions. (DOCX)

S2 Table DNA copy number aberrations in intestinal tumors from the array CGH analysis. (DOCX)

S3 Table Sequence analysis of *Apc* in intestinal tumors from B6/B6-Chr18^MSM^-F1 *Apc*^*Min/+*^ mice. (DOCX)

S1 Fig *Apc* genomic region analyzed in this study. Nucleotide positions are numbered relative to the transcription start site of *Apc* (NM_007462). Capital letters represent CpG islands obtained from the UCSC Genome Browser (http://genome.ucsc.edu/). The CpG sites are highlighted in red. Primer pairs (boxed sequences) were designed using MethPrimer (http://www.urogene.org/cgi-bin/methprimer/methprimer.cgi). Arrows indicate the direction from 5’ to 3’. Due to the relative length of this CpG island, it was split into two segments, and bisulfite sequencing was performed for each segment.
(TIF)

S2 Fig Analysis of LOH of Chr18 in mouse intestinal tumors. Each row shows the LOH of one microsatellite marker on Chr18, and each column shows the data for one intestinal tumor. Open circles indicate the loss of either the wild-type or Min alleles, and filled circles indicate the retention of both alleles. Based on the LOH results, each tumor was categorized as 0 Gy Unidentified, 2 Gy Unidentified, or 2 Gy Deletion.
(TIF)

S3 Fig Analysis of *Apc* mRNA expression in the small intestinal tumors of B6/B6-Chr18^MSM^-F1 Apc^*Min/+*^ mice. Quantitative PCR was performed using *Gapdh* as an endogenous control. Each point represents *Apc* mRNA level in an individual tumor (0 Gy Unidentified, n = 14; 2 Gy Unidentified, n = 15; 2 Gy Deletion, n = 6; normal intestinal tissue, n = 4). Primers were set to span exons 7 and 8 (A) and exons 16 and 17 (B) according to the NCBI database (NM_001402727.1). The means and standard deviations are represented by bars and error bars, respectively. *Apc* expression in normal intestinal tissue was set to 1. The triangle among the 2 Gy-Unidentified data points represents *Apc* expression in Tumor ID 2-2-23, in which a deletion comprised approximately 3.5 kbp at the end of the last exon of *Apc*. ** p < 0.01, *** p < 0.001 versus normal intestinal tissue group.
(TIF)

S4 Fig Bisulfite sequencing of the sites corresponding to human *APC* promoter 1A in small intestinal tumors of B6/B6-Chr18^MSM^-F1 *Apc*^*Min/+*^ mice. Methylation status of the 21 CpG dinucleotides in the promoter region of *Apc*. Each circle represents a CpG site in the genomic DNA sequence and each row of circles represents the analysis of a single cloned allele. Closed circles: methylated CpG dinucleotides; open circles: unmethylated CpG dinucleotides.
(TIF)

## References

[1] Ozasa K, Shimizu Y, Suyama A, Kasagi F, Soda M, Grant EJ, et al. Studies of the mortality of atomic bomb survivors, Report 14, 1950–2003: an overview of cancer and noncancer diseases. Radiat Res. 2012;177(3):229–43. Epub 2011/12/17. doi: 10.1667/rr2629.1 . 22171960

[2] Little JB. Radiation carcinogenesis. Carcinogenesis. 2000;21(3):397–404. doi: 10.1093/carcin/21.3.397 . 10688860

[3] Hamatani K, Eguchi H, Ito R, Mukai M, Takahashi K, Taga M, et al. RET/PTC rearrangements preferentially occurred in papillary thyroid cancer among atomic bomb survivors exposed to high radiation dose. Cancer Res. 2008;68(17):7176–82. doi: 10.1158/0008-5472.CAN-08-0293 . 18757433

[4] Inoue T, Kokubo T, Daino K, Yanagihara H, Watanabe F, Tsuruoka C, et al. Interstitial chromosomal deletion of the tuberous sclerosis complex 2 locus is a signature for radiation-associated renal tumors in Eker rats. Cancer Sci. 2020;111(3):840–8. Epub 20200203. doi: 10.1111/cas.14307 . 31925975 PMC7060461

[5] Tsuruoka C, Blyth BJ, Morioka T, Kaminishi M, Shinagawa M, Shimada Y, et al. Sensitive Detection of Radiation-Induced Medulloblastomas after Acute or Protracted Gamma-Ray Exposures in Ptch1 Heterozygous Mice Using a Radiation-Specific Molecular Signature. Radiat Res. 2016;186(4):407–14. Epub 20160930. doi: 10.1667/RR14499.1 . 27690174

[6] Tsuruoka C, Kaminishi M, Shinagawa M, Shang Y, Amasaki Y, Shimada Y, et al. High Relative Biological Effectiveness of 2 MeV Fast Neutrons for Induction of Medulloblastoma in Ptch1+/- Mice with Radiation-specific Deletion on Chromosome 13. Radiat Res. 2021;196(2):225–34. doi: 10.1667/RADE-20-00025.1 . 34046685

[7] Yamada Y, Mori H. Multistep carcinogenesis of the colon in Apc(Min/+) mouse. Cancer Sci. 2007;98(1):6–10. doi: 10.1111/j.1349-7006.2006.00348.x . 17052257 PMC11159231

[8] Sasatani M, Shimura T, Doi K, Zaharieva EK, Li J, Iizuka D, et al. Morphology dynamics in intestinal crypt during postnatal development affect age-dependent susceptibility to radiation-induced intestinal tumorigenesis in ApcMin/+ mice; possible mechanisms of radiation tumorigenesis. Carcinogenesis. in press. doi: 10.1093/carcin/bgac100 36546734 PMC10183640

[9] Iizuka D, Izumi S, Suzuki F, Kamiya K. Analysis of a lectin microarray identifies altered sialylation of mouse serum glycoproteins induced by whole-body radiation exposure. J Radiat Res. 2019;60(2):189–96. doi: 10.1093/jrr/rry100 . 30521038 PMC6430252

[10] Livak KJ, Schmittgen TD. Analysis of relative gene expression data using real-time quantitative PCR and the 2(-Delta Delta C(T)) Method. Methods. 2001;25(4):402–8. doi: 10.1006/meth.2001.1262 . 11846609

[11] Daino K, Ishikawa A, Suga T, Amasaki Y, Kodama Y, Shang Y, et al. Mutational landscape of T-cell lymphoma in mice lacking the DNA mismatch repair gene Mlh1: no synergism with ionizing radiation. Carcinogenesis. 2019;40(2):216–24. doi: 10.1093/carcin/bgz013 . 30721949

[12] Koboldt DC, Zhang Q, Larson DE, Shen D, McLellan MD, Lin L, et al. VarScan 2: somatic mutation and copy number alteration discovery in cancer by exome sequencing. Genome Res. 2012;22(3):568–76. Epub 20120202. doi: 10.1101/gr.129684.111 . 22300766 PMC3290792

[13] Cingolani P, Platts A, Wang le L, Coon M, Nguyen T, Wang L, et al. A program for annotating and predicting the effects of single nucleotide polymorphisms, SnpEff: SNPs in the genome of Drosophila melanogaster strain w1118; iso-2; iso-3. Fly (Austin). 2012;6(2):80–92. doi: 10.4161/fly.19695 . 22728672 PMC3679285

[14] Boeva V, Popova T, Bleakley K, Chiche P, Cappo J, Schleiermacher G, et al. Control-FREEC: a tool for assessing copy number and allelic content using next-generation sequencing data. Bioinformatics. 2012;28(3):423–5. Epub 20111206. doi: 10.1093/bioinformatics/btr670 . 22155870 PMC3268243

[15] Nakamura N. A hypothesis: radiation carcinogenesis may result from tissue injuries and subsequent recovery processes which can act as tumor promoters and lead to an earlier onset of cancer. Br J Radiol. 2020;93(1115):20190843. Epub 20200109. doi: 10.1259/bjr.20190843 . 31860335 PMC8519633

[16] Miyoshi Y, Nagase H, Ando H, Horii A, Ichii S, Nakatsuru S, et al. Somatic mutations of the APC gene in colorectal tumors: mutation cluster region in the APC gene. Hum Mol Genet. 1992;1(4):229–33. doi: 10.1093/hmg/1.4.229 . 1338904

[17] Zeineldin M, Neufeld KL. Understanding phenotypic variation in rodent models with germline Apc mutations. Cancer Res. 2013;73(8):2389–99. Epub 20130411. doi: 10.1158/0008-5472.CAN-12-4607 . 23580574 PMC3630257

[18] Shoemaker AR, Luongo C, Moser AR, Marton LJ, Dove WF. Somatic mutational mechanisms involved in intestinal tumor formation in Min mice. Cancer Res. 1997;57(10):1999–2006. . 9157997

[19] Kaufmann A, Vogt S, Uhlhaas S, Stienen D, Kurth I, Hameister H, et al. Analysis of rare APC variants at the mRNA level: six pathogenic mutations and literature review. J Mol Diagn. 2009;11(2):131–9. Epub 20090205. doi: 10.2353/jmoldx.2009.080129 . 19196998 PMC2665862

[20] Abumrad N, Harmon C, Ibrahimi A. Membrane transport of long-chain fatty acids: evidence for a facilitated process. J Lipid Res. 1998;39(12):2309–18. . 9831619

[21] Anderson CM, Stahl A. SLC27 fatty acid transport proteins. Mol Aspects Med. 2013;34(2–3):516–28. doi: 10.1016/j.mam.2012.07.010 . 23506886 PMC3602789

[22] Yen MC, Chou SK, Kan JY, Kuo PL, Hou MF, Hsu YL. New Insight on Solute Carrier Family 27 Member 6 (SLC27A6) in Tumoral and Non-Tumoral Breast Cells. Int J Med Sci. 2019;16(3):366–75. Epub 20190124. doi: 10.7150/ijms.29946 . 30911270 PMC6428986

[23] Lopes-Ramos CM, Quackenbush J, DeMeo DL. Genome-Wide Sex and Gender Differences in Cancer. Front Oncol. 2020;10:597788. Epub 20201123. doi: 10.3389/fonc.2020.597788 . 33330090 PMC7719817

[24] Knudson AG Jr. Mutation and cancer: statistical study of retinoblastoma. Proc Natl Acad Sci U S A. 1971;68(4):820–3. doi: 10.1073/pnas.68.4.820 . 5279523 PMC389051

[25] Jopling CL. Stop that nonsense! Elife. 2014;3:e04300. Epub 20140909. doi: 10.7554/eLife.04300 . 25205670 PMC4155323

[26] Haigis KM, Hoff PD, White A, Shoemaker AR, Halberg RB, Dove WF. Tumor regionality in the mouse intestine reflects the mechanism of loss of Apc function. Proc Natl Acad Sci U S A. 2004;101(26):9769–73. Epub 20040621. doi: 10.1073/pnas.0403338101 . 15210940 PMC470749

[27] Yamaguchi K, Nagayama S, Shimizu E, Komura M, Yamaguchi R, Shibuya T, et al. Reduced expression of APC-1B but not APC-1A by the deletion of promoter 1B is responsible for familial adenomatous polyposis. Sci Rep. 2016;6:26011. Epub 20160524. doi: 10.1038/srep26011 . 27217144 PMC4877598

[28] Virmani AK, Rathi A, Sathyanarayana UG, Padar A, Huang CX, Cunnigham HT, et al. Aberrant methylation of the adenomatous polyposis coli (APC) gene promoter 1A in breast and lung carcinomas. Clin Cancer Res. 2001;7(7):1998–2004. . 11448917

[29] Nagel R, le Sage C, Diosdado B, van der Waal M, Oude Vrielink JA, Bolijn A, et al. Regulation of the adenomatous polyposis coli gene by the miR-135 family in colorectal cancer. Cancer Res. 2008;68(14):5795–802. doi: 10.1158/0008-5472.CAN-08-0951 . 18632633

[30] Morioka T, Yamazaki S, Yanagihara H, Sunaoshi M, Kaminishi M, Kakinuma S. Calorie Restriction Suppresses the Progression of Radiation-Induced Intestinal Tumours in C3B6F1 Apc (Min/+) Mice. Anticancer Res. 2021;41(3):1365–75. doi: 10.21873/anticanres.14894 . 33788728

[31] McCart AE, Vickaryous NK, Silver A. Apc mice: models, modifiers and mutants. Pathol Res Pract. 2008;204(7):479–90. Epub 20080605. doi: 10.1016/j.prp.2008.03.004 . 18538487

