# Supplementary material for: Newly discovered genomic mutation patterns in radiation-induced small intestinal tumors of ApcMin/+ mice
Source: PLoS One. 2023 Oct 12;18(10):e0292643. doi: 10.1371/journal.pone.0292643 (PMC10569626; doi:10.1371/journal.pone.0292643)
Supplement: S1 Table — (DOCX) [file pone.0292643.s001.docx]

S1 Table. Primers and PCR conditions.

(I) Sodium bisulfite DNA sequencing

| Name | Forward primer (5' to 3') | Reverse primer (5' to 3') | Initial denaturing temperature (°C) and time (min) | | Denaturing temperature (°C) and time (sec) | | Annealing temperature (°C) and time (sec) | | Extension temperature (°C) and time (sec) | | PCR cycles | Final extension temperature (°C) and time (min) | |
| --- | --- | --- | --- | --- | --- | --- | --- | --- | --- | --- | --- | --- | --- |
| CpG island 1 | GTAGAGGTAGGGTATAGGTTGTTG | CTAAAAAAACCACTCCTCACTCC | 94 | 2 | 94 | 30 | 60 | 30 | 72 | 30 | 40 | 72 | 2 |
| CpG island 2 | GGAGTGAGGAGTGGTTTTTTTAG | ATCCTCCCAAAACTCAAATTTTAAT | - | - | 98 | 10 | 59 | 30 | 72 | 60 | 40 | - | - |

(II) Quantitative RT-PCR

| Name | Forward primer (5' to 3') | Reverse primer (5' to 3') | Initial denaturing temperature (°C) and time (sec) | | Denaturing temperature (°C) and time (sec) | | Annealing/extension temperature (°C)  and time (sec) | | PCR cycles |
| --- | --- | --- | --- | --- | --- | --- | --- | --- | --- |
| *Apc (exon2 and exon3)* | CGACAAGAGCTAGAAGATAATTCCA | TCAATACTTCCCTGTAGCTGCTT | 95 | 60 | 95 | 15 | 60 | 60 | 45 |
| *Apc (exon7 and exon8)* | GCGGAAGCGGAGAGGTCATC | CTACTGGAGGCTGCGGTGTT |  |  |  |  |  |  |  |
| *Apc (exon16 and exon17)* | AACGAAGACCACAGGCAAATCC | CCATGTCCCACAAGGCTTCC |  |  |  |  |  |  |  |
| *Gapdh* | GTCAGCAATGCATCCTGCA | GTGGTCATGAGCCCTTCCA |  |  |  |  |  |  |  |
