# Supplementary material for: Newly discovered genomic mutation patterns in radiation-induced small intestinal tumors of ApcMin/+ mice
Source: PLoS One. 2023 Oct 12;18(10):e0292643. doi: 10.1371/journal.pone.0292643 (PMC10569626; doi:10.1371/journal.pone.0292643)
Supplement: S2 Table — (DOCX) [file pone.0292643.s002.docx]

S5 Table. DNA copy number aberrations of intestinal tumors in an array CGH analysis.

|  | Tumor ID | Cytoband | Probe no. | Size (kbp) | log_2_ ratio | | Number of genes involved* |
| --- | --- | --- | --- | --- | --- | --- | --- |
|  |  |  |  |  | Gain | Loss |  |
| 0 Gy Unidentified | 0-1-3 | 18qD3 | 31 | 46.0 |  | -0.59 | 1 |
|  |  | XqA1.1qF5 | 47 | 158567.7 | 0.54 | — | 825 |
|  | 0-1-11 | 18qD3 | 32 | 47.5 | — | -0.57 | 1 |
|  |  | XqA1.1qF5 | 46 | 156844.8 | 0.58 | — | 819 |
|  | 0-1-12 | 18qD3 | 32 | 47.5 | — | -0.56 | 1 |
|  |  | XqA1.1qF5 | 47 | 158567.7 | 0.64 | — | 825 |
|  | 0-2-4 | XqA1.1qF5 | 47 | 158567.7 | — | -0.39 | 825 |
|  | 0-2-12 | XqA1.1qF5 | 47 | 158567.7 | — | -0.35 | 825 |
|  | 0-2-24 | XqA7.3qF1 | 28 | 61681.5 | — | -0.59 | 272 |
| 2 Gy Unidentified | 2-2-5 | XqA7.3qF1 | 29 | 64263.6 | — | -0.67 | 290 |
|  | 2-2-23 | XqA7.3qF1 | 28 | 61681.5 | — | -0.62 | 272 |
|  | 2-2-39 | XqA1.1qF5 | 47 | 158567.7 | — | -0.35 | 825 |
| 2 Gy Deletion | 2-2-19 | 18qB1qD2 | 12790 | 21470.9 | — | -0.34 | 212 |
|  |  | XqA1.2qF5 | 45 | 148514.1 | — | -0.29 | 735 |
|  | 2-2-26 | 18qA2qB2 | 5273 | 8849.9 | — | -0.29 | 58 |
|  |  | XqA1.1qF5 | 47 | 158567.7 | — | -0.37 | 825 |
|  | 2-2-29 | 18qA1qB3 | 17216 | 29282.9 | — | -0.34 | 212 |
|  | 2-2-32 | 18qA2qC | 13606 | 22713.4 | — | -0.26 | 211 |
|  | 2-2-43 | 18qA2qB3 | 7346 | 12358.4 | — | -0.38 | 111 |
|  |  | XqA1.1qF5 | 47 | 158567.7 | — | -0.36 | 825 |
|  | 2-2-56 | 18qA2qB2 | 7215 | 11944.0 | — | -0.39 | 71 |
|  |  | XqA1.1qF5 | 47 | 158567.7 | — | -0.34 | 825 |

*The number of genes involved in each aberration was counted using Agilent Genomic Workbench software.
