# Supplementary material for: Newly discovered genomic mutation patterns in radiation-induced small intestinal tumors of ApcMin/+ mice
Source: PLoS One. 2023 Oct 12;18(10):e0292643. doi: 10.1371/journal.pone.0292643 (PMC10569626; doi:10.1371/journal.pone.0292643)
Supplement: S3 Table — (DOCX) [file pone.0292643.s003.docx]

S6 Table. Sequencing analysis of *Apc* in intestinal tumors from B6/B6-Chr18^MSM^-F1 *Apc^Min/+^* mice.

|  | Tumor ID | Position in chr.18* | Ref. Seq | Alt. Seq | Variant allele frequency (%) | HGVSc^†^ | Annotation^‡^ | HGVSp^§^ | Location in Mus musculus APC, WNT signaling pathway regulator (Apc), transcript variant 3 (ENSMUST00000079362.11) |
| --- | --- | --- | --- | --- | --- | --- | --- | --- | --- |
| 0 Gy Unidentified | 0-1-3 | 34240682 | AT | A | 1.41 | c.-19+19414delT | intron_variant |  | intron1 |
|  |  | 34241193 | CT | C | 1.22 | c.-18-19804delT | intron_variant |  | intron1 |
|  |  | 34243490 | TG | T | 3.12 | c.-18-17513delG | intron_variant |  | intron1 |
|  |  | 34268081 | A | G | 1.00 | c.136-217A>G | intron_variant |  | intron2 |
|  |  | 34275177 | GA | G | 7.14 | c.526-1377delA | intron_variant |  | intron5 |
|  | 0-1-11 | 34275177 | GA | G | 4.76 | c.526-1377delA | intron_variant |  | intron5 |
|  |  | 34322360 | A | G | 1.01 | c.*3779A>G | downstream_gene_variant |  | downstream of 3’UTR |
|  | 0-1-12 | 34240641 | T | C | 3.08 | c.-19+19365T>C | intron_variant |  | intron1 |
|  |  | 34243490 | TG | T | 1.57 | c.-18-17513delG | intron_variant |  | intron1 |
|  |  | 34298758 | G | A | 6.39 | c.1263G>A | stop_gained | p.Trp421* | exon10 |
|  |  | 34308125 | AAAG | A | 3.51 | c.1737+1749_1737+1751delGAA | intron_variant |  | intron14 |
|  | 0-2-4 | ND |  |  |  |  |  |  |  |
|  | 0-2-14 | 34271959 | C | T | 30.04 | c.417-480C>T | intron_variant |  | intron4 |
|  |  | 34275177 | GA | G | 4.76 | c.526-1377delA | intron_variant |  | intron5 |
|  | 0-2-24 | 34243490 | TG | T | 1.14 | c.-18-17513delG | intron_variant |  | intron1 |
|  |  | 34312033 | C | T | 30.39 | c.1981C>T | stop_gained | p.Gln661* | exon16 |
| 2 Gy Unidentified | 2-2-5 | 34239701 | AT | A | 2.63 | c.-19+18435delT | intron_variant |  | intron1 |
|  | 2-2-11 | 34275177 | GA | G | 3.57 | c.526-1377delA | intron_variant |  | intron5 |
|  | 2-2-20 | 34239701 | AT | A | 3.03 | c.-19+18435delT | intron_variant |  | intron1 |
|  |  | 34243490 | TG | T | 1.82 | c.-18-17513delG | intron_variant |  | intron1 |
|  | 2-2-23 | 34239701 | AT | A | 3.17 | c.-19+18435delT | intron_variant |  | intron1 |
|  |  | 34240682 | AT | A | 1.85 | c.-19+19414delT | intron_variant |  | intron1 |
|  |  | 34267972 | A | G | 1.4 | c.136-326A>G | intron_variant |  | intron2 |
|  |  | 34302316 | G | T | 29.27 | c.1402+2255G>T | intron_variant |  | intron11 |
|  |  | 34314404 | T | C | 1.34 | c.4352T>C | missense_variant | p.Val1451Ala | exon16 |
|  | 2-2-38 | 34240682 | AT | A | 1.17 | c.-19+19414delT | intron_variant |  | intron1 |
|  |  | 34307326 | GGT | G | 4.17 | c.1737+949_1737+950delTG | intron_variant |  | intron14 |
|  |  | 34308125 | AAAG | A | 4.08 | c.1737+1749_1737+1751delGAA | intron_variant |  | intron14 |
|  | 2-2-39 | 34239701 | AT | A | 3.64 | c.-19+18435delT, | intron_variant |  | intron1 |
|  |  | 34243490 | TG | T | 3.27 | c.-18-17513delG | intron_variant |  | intron1 |
|  |  | 34308125 | AAAG | A | 3.45 | c.1737+1749_1737+1751delGAA | intron_variant |  | intron14 |
|  |  | 34312841 | C | G | 21.59 | c.2789C>G | stop_gained | p.Ser930* | exon16 |
|  | 2-2-42 | 34279314 | GT | G | 21.3 | c.686delT | frameshift_variant | p.Val229fs | exon7 |
|  |  | 34308125 | AAAG | A | 3.12 | c.1737+1749_1737+1751delGAA | intron_variant |  | intron14 |

All genomic locations were unified in the corresponding mouse mm10 genomic coordinates.

^†^The “intron_variant” does not contain mutations in the splice acceptor/donor site.

^‡^HGVS (Human Genome Variation Society) coding sequence name.

^§^HGVS protein sequence name.
