# Supplementary figures and images for: Newly discovered genomic mutation patterns in radiation-induced small intestinal tumors of ApcMin/+ mice
Source: PLoS One. 2023 Oct 12;18(10):e0292643. doi: 10.1371/journal.pone.0292643 (PMC10569626; doi:10.1371/journal.pone.0292643)

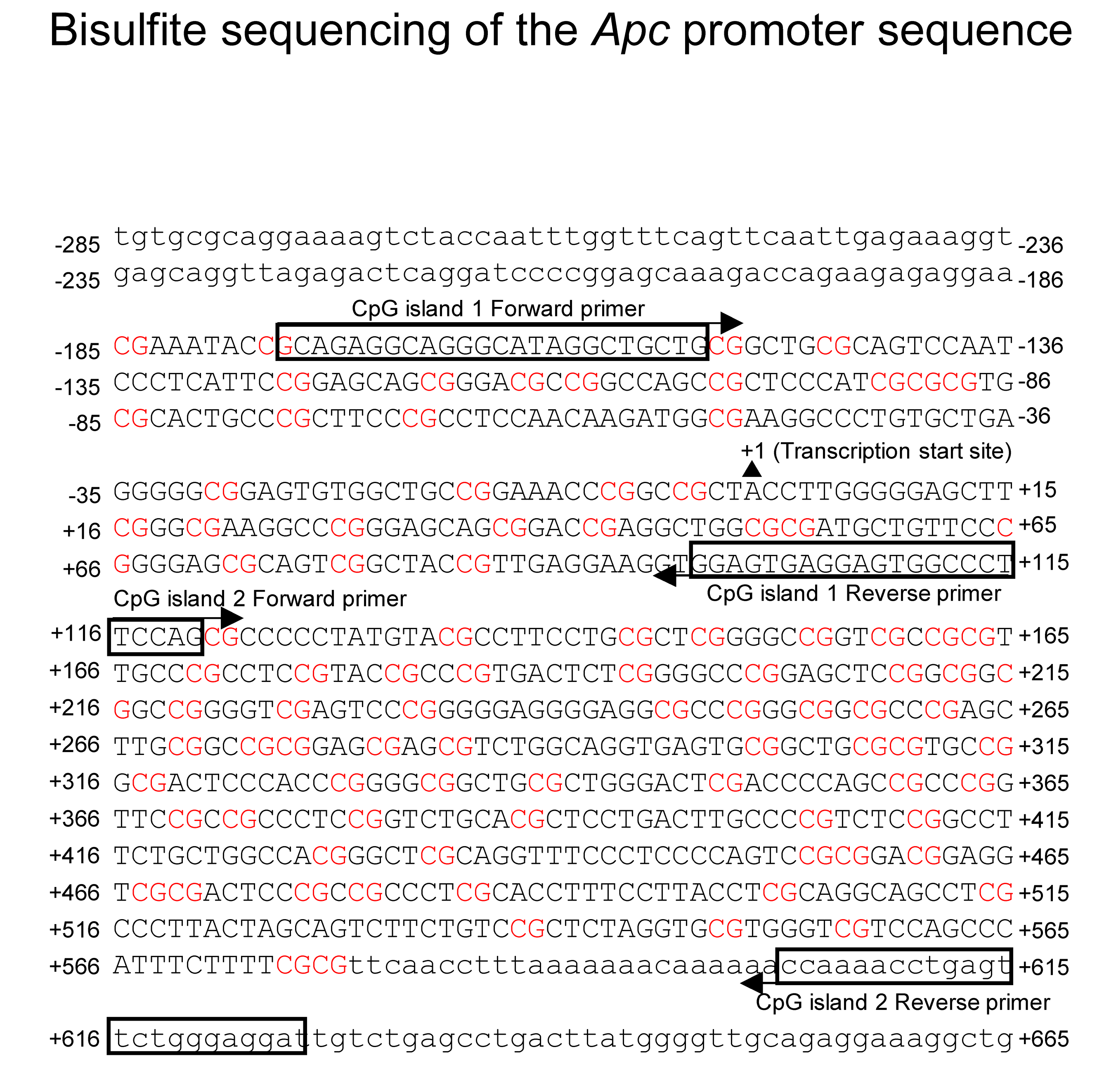

Supplement: S1 Fig — Nucleotide positions are numbered relative to the transcription start site of Apc (NM_007462). Capital letters represent CpG islands obtained from the UCSC Genome Browser (http://genome.ucsc.edu/). The CpG sites are highlighted in red. Primer pairs (boxed sequences) were designed using MethPrimer (http://www.urogene.org/cgi-bin/methprimer/methprimer.cgi). Arrows indicate the direction from 5’ to 3’. Due to the relative length of this CpG island, it was split into two segments, and bisulfite sequencing was performed for each segment. (TIF) [file pone.0292643.s004.tif]

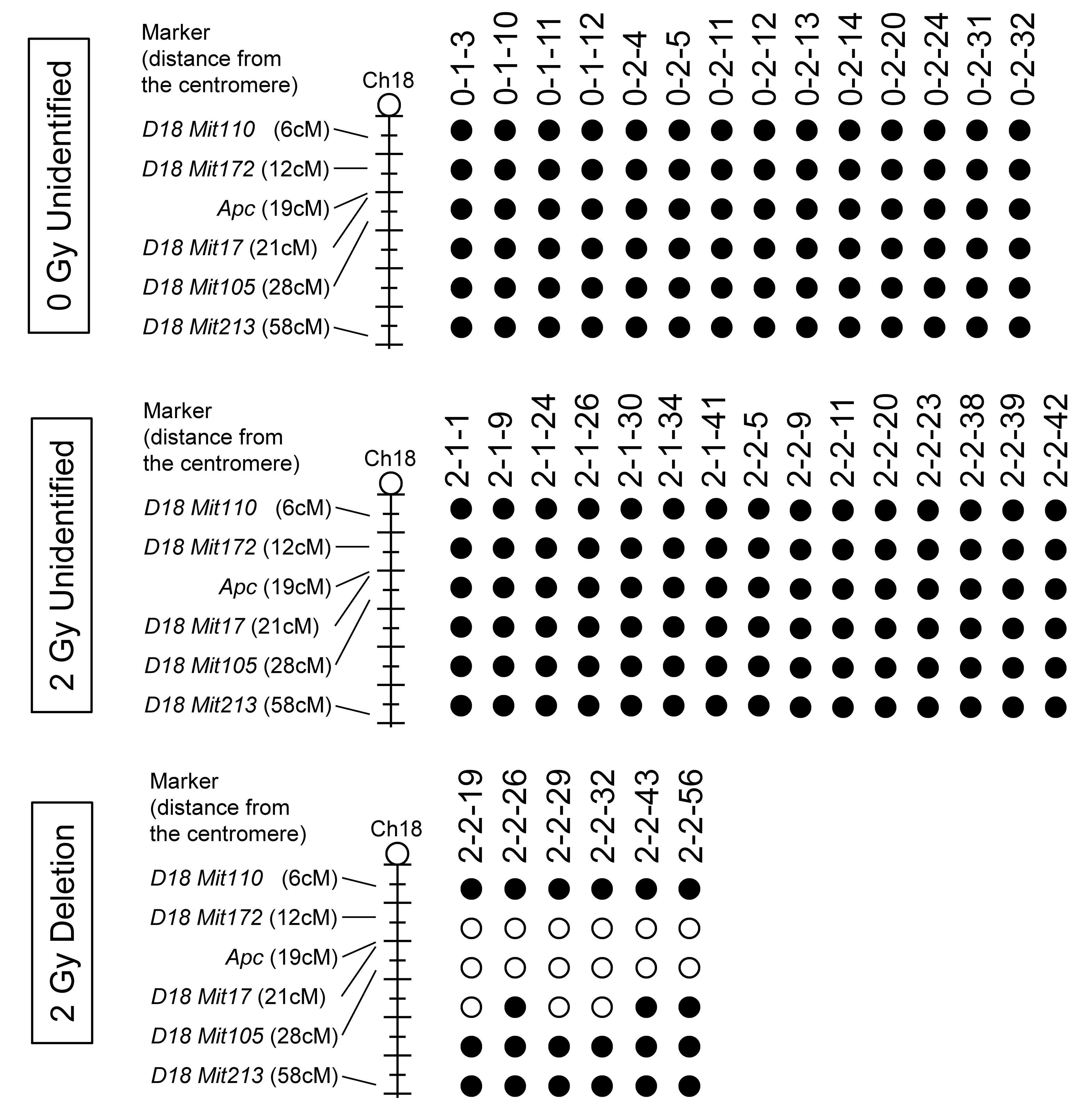

Supplement: S2 Fig — Each row shows the LOH of one microsatellite marker on Chr18, and each column shows the data for one intestinal tumor. Open circles indicate the loss of either the wild-type or Min alleles, and filled circles indicate the retention of both alleles. Based on the LOH results, each tumor was categorized as 0 Gy Unidentified, 2 Gy Unidentified, or 2 Gy Deletion. (TIF) [file pone.0292643.s005.tif]

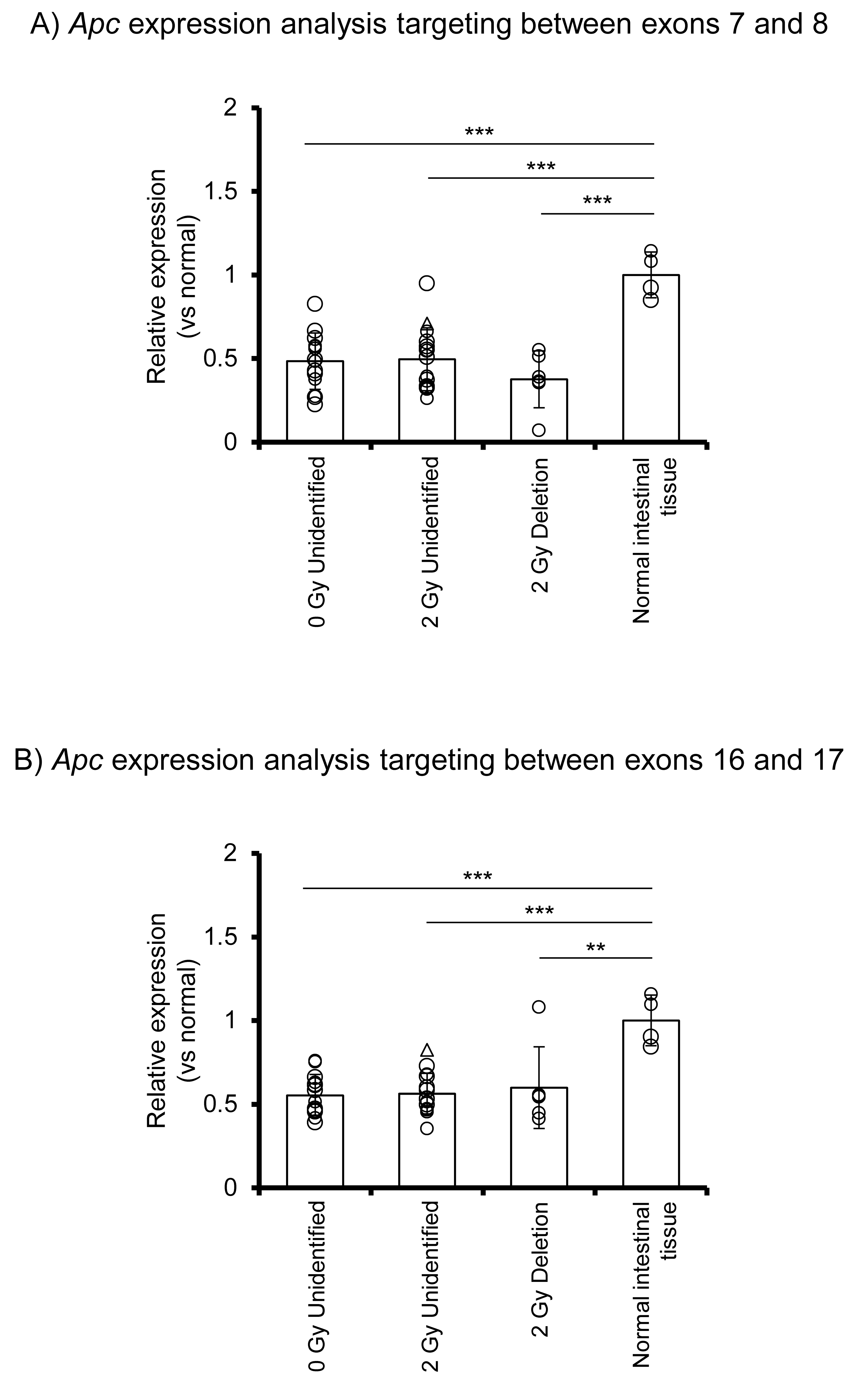

Supplement: S3 Fig — Quantitative PCR was performed using Gapdh as an endogenous control. Each point represents Apc mRNA level in an individual tumor (0 Gy Unidentified, n = 14; 2 Gy Unidentified, n = 15; 2 Gy Deletion, n = 6; normal intestinal tissue, n = 4). Primers were set to span exons 7 and 8 (A) and exons 16 and 17 (B) according to the NCBI database (NM_001402727.1). The means and standard deviations are represented by bars and error bars, respectively. Apc expression in normal intestinal tissue was set to 1. The triangle among the 2 Gy-Unidentified data points represents Apc expression in Tumor ID 2-2-23, in which a deletion comprised approximately 3.5 kbp at the end of the last exon of Apc. ** p < 0.01, *** p < 0.001 versus normal intestinal tissue group. (TIF) [file pone.0292643.s006.tif]

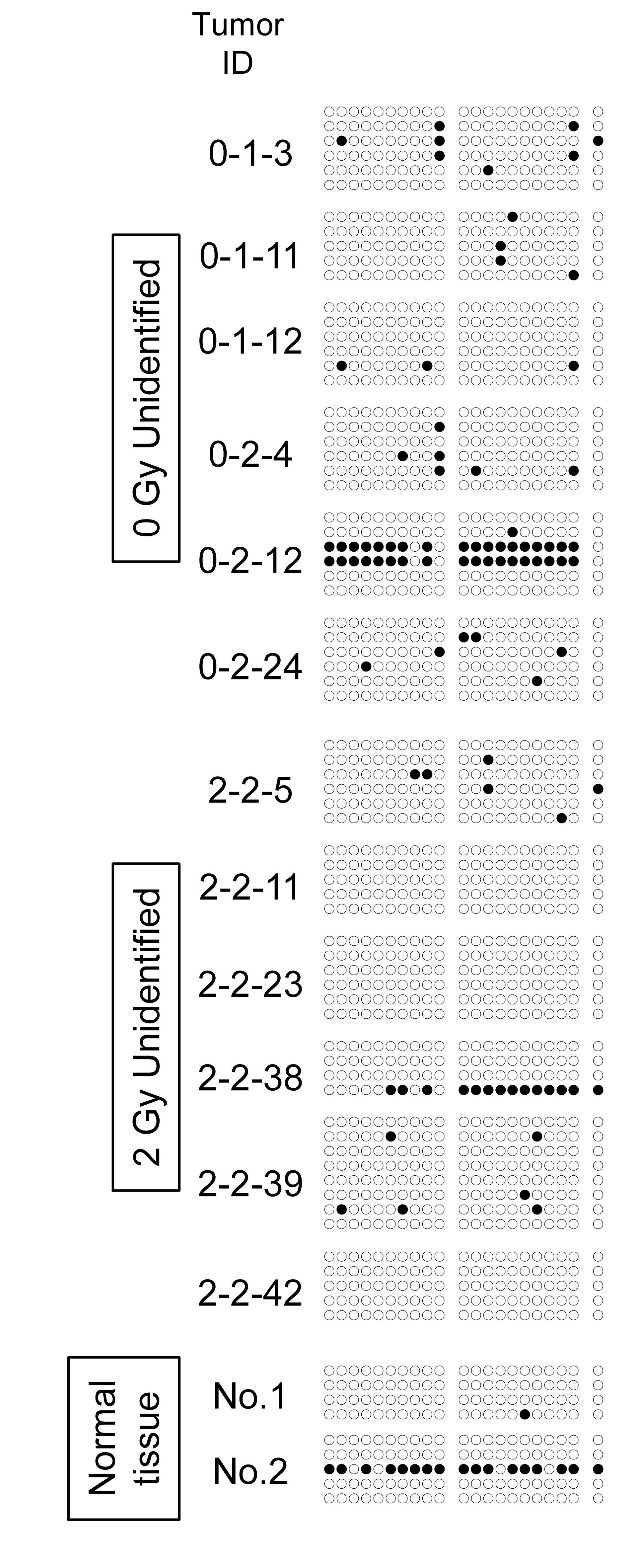

Supplement: S4 Fig — Methylation status of the 21 CpG dinucleotides in the promoter region of Apc. Each circle represents a CpG site in the genomic DNA sequence and each row of circles represents the analysis of a single cloned allele. Closed circles: methylated CpG dinucleotides; open circles: unmethylated CpG dinucleotides. (TIF) [file pone.0292643.s007.tif]
